# To what extent do structural changes in catalytic metal sites affect enzyme function?

**DOI:** 10.1016/j.jinorgbio.2017.11.002

**Published:** 2018-02

**Authors:** Yana Valasatava, Antonio Rosato, Nicholas Furnham, Janet M. Thornton, Claudia Andreini

**Affiliations:** a Magnetic Resonance Center, University of Florence, 50019 Sesto Fiorentino, Italy; b Department of Chemistry, University of Florence, 50019 Sesto Fiorentino, Italy; c Department of Pathogen Molecular Biology, London School of Hygiene and Tropical Medicine, Keppel Street, London WC1E 7HT, United Kingdom; d EMBL-European Bioinformatics Institute, Wellcome Trust Genome Campus, Hinxton, Cambridge CB10 1SD, United Kingdom

**Keywords:** Metallo-proteins, Bioinorganic chemistry, Iron, Zinc, Copper, Magnesium, Evolution, Enzymes

## Abstract

About half of known enzymatic reactions involve metals. Enzymes belonging to the same superfamily often evolve to catalyze different reactions on the same structural scaffold. The work presented here investigates how functional differentiation, within superfamilies that contain metalloenzymes, relates to structural changes at the catalytic metal site. In general, when the catalytic metal site is unchanged across the enzymes of a superfamily, the functional differentiation within the superfamily tends to be low and the mechanism conserved. Conversely, all types of structural changes in the metal binding site are observed for superfamilies with high functional differentiation. Overall, the catalytic role of the metal ions appears to be one of the most conserved features of the enzyme mechanism within metalloenzyme superfamilies. In particular, when the catalytic role of the metal ion does not involve a redox reaction (i.e. there is no exchange of electrons with the substrate), this role is almost always maintained even when the site undergoes significant structural changes. In these enzymes, functional diversification is most often associated with modifications in the surrounding protein matrix, which has changed so much that the enzyme chemistry is significantly altered. On the other hand, in more than 50% of the examples where the metal has a redox role in catalysis, changes at the metal site modify its catalytic role. Further, we find that there are no examples in our dataset where metal sites with a redox role are lost during evolution.

**Synopsis:**

In this paper we investigate how functional diversity within superfamilies of metalloenzymes relates to structural changes at the catalytic metal site. Evolution tends to strictly conserve the metal site. When changes occur, they do not modify the catalytic role of non-redox metals whereas they affect the role of redox-active metals.

## Highlights

• Evolution tends to strictly conserve the site of the catalytic metal ion.
• Even when the site changes, the role of not redox active metals is preserved.
• Site changes in redox-active metals often change their role in catalysis.
• The role of the metal in the catalysis evolves slower than the overall enzyme chemistry.
• Redox-active metals are never lost in the evolution.

## Graphical abstract

**Figure:**
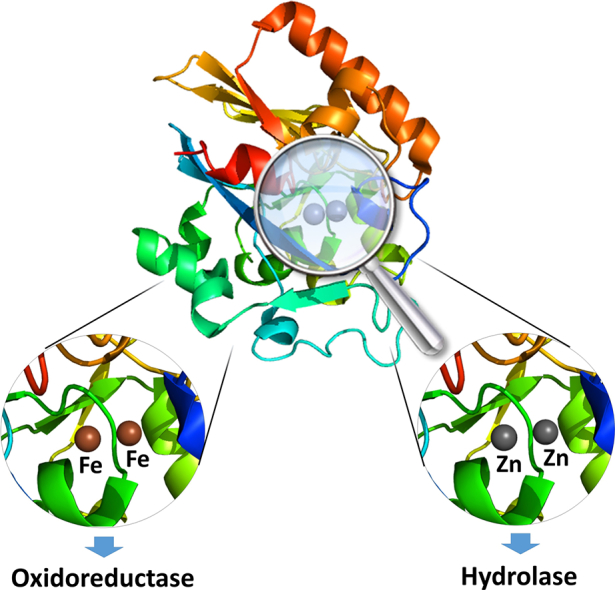

## 1 Introduction

Enzymes are the proteins responsible for the catalysis of chemical reactions in biological systems and as such are central to life. A vast area of biochemistry seeks to elucidate their reaction mechanisms and the subtle structure-function relationships underlying those mechanisms. The knowledge gained has many applications in medicine and agriculture [1].

Studies aimed at finding general principles underlying enzyme mechanisms typically rely on the comparative analysis of many different enzymes, and are thus closely related to the study of enzyme evolution. The central idea of these approaches is that enzymes with a common ancestor can be grouped into families and superfamilies based on sequence and structure similarity [2], [3], [4], [5], [6], [7]. Subsequently, the comparison of enzymes within and across groups sheds light on how changes in the sequence and/or in the structure are related to changes in enzyme function [8], [9], [10], [11], [12], [13]. Such analyses normally focus on how mutations of amino acids that are directly involved in the catalytic reaction affect the function of the enzyme. In metalloenzymes, which represent approximately 40% of all enzymes [14], the catalytic activity of the enzyme depends also on at least one metal-containing cofactor located in the active site. The complexity of these cofactors may range from an individual ion to highly elaborate polymetallic clusters such as the FeMoco cluster of nitrogenases (this cofactor is a cluster with composition Fe_7_MoS_9_C). In these enzymes, changes in function during evolution can also be due to changes in the metal cofactor [15]. Furthermore, functional changes can also result from changes in metal coordination by the protein matrix, since the chemical properties of certain metal ions, and thus their catalytic activity, can be finely modulated by the properties of their local environment [16], [17], [18], [19]. As a consequence, the evolutionary study of metal-dependent enzymes requires a further level of analysis that correlates the function not only with the sequence but also with the metal ion and its environment.

Based on their knowledge of metal chemistry, bioinorganic chemists [20] have suggested several principles governing metal-assisted catalysis, such as the idea that zinc ions activate water molecules acting as nucleophiles [21]. Nevertheless, to our knowledge, there is no available study systematically investigating the effect of specific changes in the metal-containing active site on the function of metalloenzymes. In this work, we address this issue by combining data from the FunTree database [13], which brings together sequence, structure, phylogenetic, chemical and mechanistic information for structurally defined enzyme superfamilies, with other resources specifically designed for the study of metalloenzymes. The resources include the Metal-MACiE database (the Metal-dependent subset of the database of Mechanism, Annotation and Classification in Enzymes) [22], which contains a manually curated annotation on metalloenzyme mechanisms, and MetalPDB (the Metal-binding subset of the Protein Data Bank) [23], which collects structural information on metal sites [17], [24]. The results of the analysis provides for the first time a confirmation, underpinned by a large and diverse data set, of assumptions and intuitions of bioinorganic chemists [25], [26], [27], thereby strengthening and widening our understanding of the function and evolution of metalloenzymes and catalysis in general.

## 2 Materials and methods

**Fig. 1:**
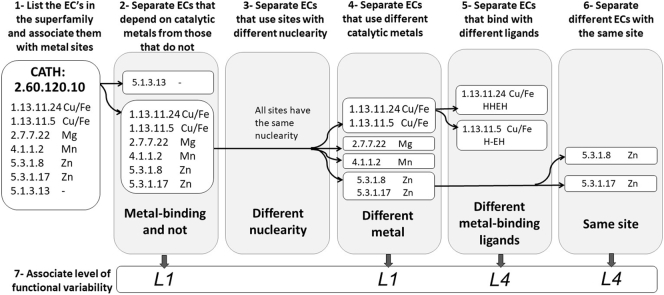
Pipeline to separate a given CATH superfamily into defined subgroups based on subsequent splitting events. The occurrence of splitting events (steps 1–6) is evaluated hierarchically. The level of functional differentiation (defined as the highest level at which the EC numbers changed for any possible pair of superfamily members in the different subgroups created) is assigned to each splitting event at the end of the procedure (step 7). It is important to note that this pipeline does not necessarily capture the evolutionary history of the family and its members.

We extracted all the catalytic metal sites with an available 3D structure (i.e. those directly involved in a catalytic reaction) from the Metal-MACiE database. All metal ions in Metal-MACiE are manually annotated by literature analysis, so the dataset is rigorously curated. Hereafter, Metal-MACiE entries will be labelled as MM: followed by the corresponding database identifier (e.g. MM:0137). The Metal-MACiE entries map to 64 CATH (Class, Architecture, Topology and Homologous superfamily classification of protein domains) [28] protein domain superfamilies. A CATH superfamily is an ensemble of homologous proteins that share a common fold and a degree of sequence similarity. All the distinct EC (Enzyme Commission) numbers within a CATH superfamily are listed and, when present, associated with a catalytic metal site (Fig. 1, step 1). EC numbers are used to describe the reaction catalyzed by an enzyme and consist of a hierarchical classification system developed and maintained by the Enzyme Commission. It is a four-level descriptor (in the form L1.L2.L3.L4), that hierarchically designates the general reaction type (denoted by the first digit i.e. L1) and further defines the mechanism, substrates, products and cofactors with the series of the three numbers that follow. To associate a catalytic metal site with each EC number we map the positions of the protein residues which coordinate the catalytic metal ion (metal-binding ligands, hereafter) onto the multiple sequence alignments obtained from the FunTree database [13]. When the representative sequences presented in the FunTree alignment of a given superfamily did not include a metalloenzyme of interest, we added the sequence of the Metal-MACiE entry to the FunTree alignment using the program TM-ALIGN [29]. Each sequence in the alignment was labelled as “metal-binding” if at least 50% of the metal-binding ligands are conserved. For metal-binding sites with only two ligands, the label was assigned to sequences conserving the entire site.

From step 2 to step 6 of our pipeline, the information obtained on the catalytic metal sites of enzymes was exploited to split the list of EC numbers within each superfamily on the basis of their metal-binding properties. In particular, in the second step (Fig. 1, step 2) we separated EC numbers dependent on a catalytic metal ion from those that are independent and thus have lost the metal-binding site. The latter were not analyzed further. In the third step of our pipeline (Fig. 1, step 3), we separated EC numbers which use metal-binding sites with different nuclearity (i.e. the number of metal ions bound in the site). At this point, each sub-group of EC numbers contains a subset of catalytic metal-binding sites that have the same nuclearity. In the fourth step (Fig. 1, step 4), each group is further separated based on the chemical identity of the metal ion(s) in the sites. Subsequently, each subgroup of sites that have the same metal ion(s) is separated based on the identity of the metal-binding residues (Fig. 1, step 5). Finally (Fig. 1, step 6), sites that have the same metal(s) and the same metal-binding residues but differ in their associated ECs are split.

In summary, the pipeline depicted on Fig. 1 is a hierarchical procedure that separates the initial list of EC numbers in each CATH superfamily into smaller subgroups that have different metal-binding properties from each other. We called such separation a splitting event. For each split, occurring in a given superfamily, we associated its maximum functional diversity using the variation of EC numbers as a metric. The largest functional difference between two enzymes, in terms of the reactions catalyzed, occurs when their EC numbers differ at the first (primary) level (e.g. 2.1.1.1 vs. 3.1.2.2). This progresses to the fourth level that differentiates the minor functional details such as substrate specificity. There is no correlation between the differences in the reactions catalyzed and the variation within a given level of EC number; so, for example, EC number 2.1.1.1 is no more similar to 2.1.1.2 than it is to 2.1.1.10. Therefore, the functional diversity is defined as the highest level at which the EC numbers for any possible pair of sites from two subgroups differ (Fig. 1, step 7). This includes the following four levels of impact, for a change at each level of EC number, (moving from the smallest to the largest changes in function):

• Diversity at level 4 (change of serial number, usually associated with a different substrate), labelled as L4
• Diversity at level 3 (this has various meanings depending on the primary class), labelled as L3
• Diversity at level 2 (a different sub-class, usually representing a bond being broken or formed), labelled as L2
• Diversity at level 1 (a different general reaction type), labelled as L1

The final analysis involved manual examination of functional and structural details extracted from various sources, which typically included: the publications describing the structures deposited in the PDB (Protein Data Bank) [30]; MetalPDB [23]; Metal-MACiE [22]; PDBSprotEC [31]; BRENDA [32]. To compare metal-binding sites of metalloenzymes with known structure we used the MetalS^2^ tool (Metal Sites Superposition tool) [33]; to identify metal-binding sites structurally similar to a given metal-binding site we searched the MetalPDB database using the MetalS^3^ (Metal Sites Similarity Search tool) tool [34].

## 3 Results

### 3.1 A global view of structural, functional diversification in metalloenzymes

From the Metal-MACiE resource we extracted a total of 106 catalytic metals included in 3D structures. The metalloenzymes containing these sites mapped to 64 CATH [28] superfamilies. Eight of these superfamilies include enzymes with just one EC number associated (and thus just one enzyme function), so there were no splitting events within them (Supplementary Table S1). The catalytic metal site is conserved in all the members of these superfamilies, with one exception - GTP cyclohydrolase I (GYCH-I). The GYCH-IA enzyme is present in all kingdoms of life and depends on zinc(II), whereas the prokaryotic-specific GYCH-IB has the same fold, but is expressed under zinc starvation and uses other divalent cations to perform the same chemistry [35].

**Fig. 2:**
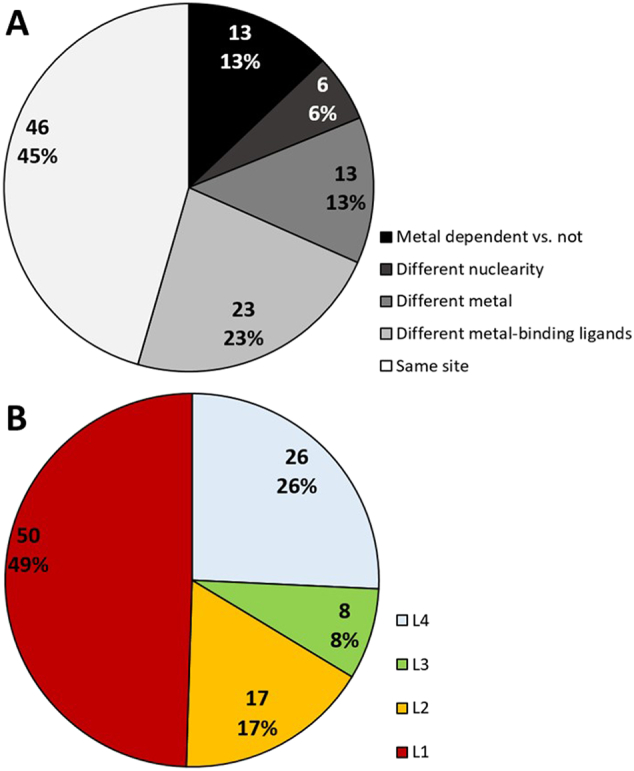
Separation of the 101 splitting events based on (A) the type of metal site changes and (B) the maximum functional differentiation.

The remaining 56 superfamilies include metal-dependent enzymes with different EC numbers, and thus correspond to examples where the same protein fold is adapted to perform different catalytic functions. The application of our pipeline to these superfamilies lead to the identification of 101 splitting events (Fig. 1, see Materials and methods). The most common splitting event that occurs in our dataset (i.e. 45% of cases; 46/101) is the divergence to different EC numbers that do not correspond to any change in the properties of the metal site (i.e. labelled in Fig. 2A as *Same site*). In all these cases, the functional variation is associated with changes in the protein matrix that do not affect directly the metal site. The second most frequent event is the variation in the identity and/or the number of the residues in the first coordination sphere of the metal ion (23% of cases, 23/101, labelled in the Fig. 2A as *Different metal-binding ligands*). 13% of the splitting events (i.e. 13/101) correspond to enzymes depending on different metal ions (labelled in the Fig. 2A as *Different metals*). Changes in nuclearity (i.e. the number of metal ions in the catalytic site) are comparatively less common (6%, 6/101 labelled in Fig. 2A as *Different nuclearity*). Finally, 13% of splitting events (i.e. 13/101) correspond to superfamilies that contains both enzymes which depend on metal ions and enzymes that do not use any metal ion to carry out the reaction mechanism, (i.e. labelled as *Metal dependent and metal independent*).

**Fig. 3:**
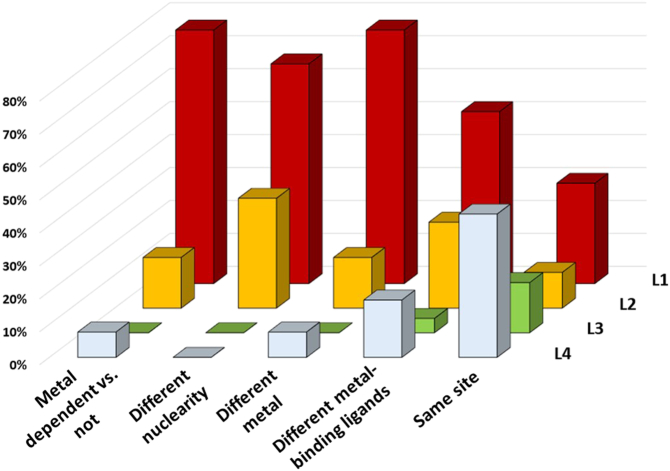
Relationship between functional differentiation and types of metal site variation.

Combining all the data, we observed a remarkable functional differentiation within superfamilies, with most superfamilies including members that differ at the primary EC level (49%, 50/101, Fig. 2B). Fig. 3 shows that conservation of the structure of the metal site is associated with lower functional differentiation (L4 in Fig. 3), while any change in the structure of the metal site is highly likely to co-occur with major changes in the function of the enzymes, i.e. members differ at the first EC level (L1 in Fig. 3).

### 3.2 An overview on the metal role changes within catalysis

To evaluate the impact of structural changes on the catalytic role of the metal ion within the catalysis we also inspect the available reaction mechanisms of different enzymes belonging to the same superfamily. Further, we classified these roles into two distinct subgroups, i.e. “redox role”, when the metal ion accepts/donates an electron during the reaction mechanism, and “Not redox role” when although participating in the reaction the metal does not modify its oxidation state (Table 1, Table 2, Table 3, Table 4).

**Table 1:** Splitting events that separate ECs that depend on metal ions from those that do not. The first column reports the CATH code of each superfamily. The second column indicates whether the role of the metal in catalysis is conserved across subgroups. The third column indicates whether the metal ion has a redox role within the catalytic mechanism. The fourth column reports the chemical identity of the metal ions. Superfamily members that do not depend on metal ions are indicated as “Apo”. EC numbers associated to enzymes that bind different metal ions e.g. in different organisms, are reported on the same line, with a list of all their metal ions separated by “/”. The fifth column reports the level of functional differentiation for the splitting event. The sixth column reports the different EC numbers within the subgroups formed by the present splitting event. Note that subgroups containing multiple ECs will be further separated in the next tables following the pipeline of Fig. 1. The last column reports a short mention of the role(s) of metal(s) within the catalytic mechanism. In this table, the mechanistic details given in the last column refer only to the metal-dependent superfamily members.

CATH code	Metal role conserved	Redox role	Ion(s)	EC diversity	EC(s)	Mechanism
3.20.20.190	No	No	Ca	L4	3.1.4.11 3.1.4.46	Stabilizes charges and increases electrophilicity of substrate
			Apo		3.1.4.10	
3.40.50.720	No	No	Mg	L2	1.1.1.38 1.1.1.39 1.1.1.40	Stabilizes charges and increases substrate acidity
			Apo		1.4.1.2 1.4.1.3 1.2.1.12	
3.90.180.10	No	No	Zn	L2	1.1.1.1 1.1.1.284 1.1.1.103 1.1.1.14	Increases substrate acidity
			Apo		1.6.5.5	
2.60.120.10	No	No	Zn/Cu/ Mg/Mn	L1	5.3.1.8 5.3.1.17 1.13.11.24 1.13.11.5 2.7.7.22 4.1.1.2	Stabilizes charges or radical intermediates
			Apo		5.1.3.13	
3.40.140.10	No	No	Zn	L1	3.5.4.5 3.5.4.2 3.5.4.12	Increases acidity and nucleophilicity of a water molecule
			Apo		2.7.4.7	
2.120.10.30	No	No	Ca	L1	1.1.5.2 3.1.1.17	Stabilizes charges and increases substrate acidity
			Apo		2.7.11.1	
2.160.20.10	No	No	Ca	L1	4.2.2.2	Stabilizes charges and increases substrate acidity
			Apo		3.1.1.11 3.2.1.15	
3.20.20.70	No	No	Mg/Zn/Fe/Co/Mn	L1	4.1.1.85 4.2.1.24 2.3.3.13 5.1.3.1 4.1.2.13 6.4.1.1 2.5.1.54	Increases substrate acidity and stabilizes charges
			Apo		1.1.1.205 1.1.2.3 2.2.1.2 4.2.1.52 5.3.1.1	
3.30.1130.10	No	No	Zn	L1	3.5.4.16	Increases acidity and nucleophilicity of a water molecule
			Apo		1.7.1.13 4.1.2.25	
3.40.50.620	No	No	Mg	L1	6.1.1.17 6.1.1.16 6.1.1.19 6.1.1.9 6.3.1.5 6.3.3.4	Stabilizes charges and increases electrophilicity of substrate
			Apo		6.3.5.2 2.7.7.3 2.7.7.4 2.7.7.18	
3.20.20.120	No	No	Mg/Mn	L1	4.2.1.11 4.2.1.113 4.2.1.40 4.2.1.6 5.5.1.1 5.1.2.2	Stabilizes charges and increases substrate acidity
			Apo		1.17.7.1	
1.10.606.10	No	No	V	L1	1.11.1.10 1.11.1.18	Stabilizes charges and increases electrophilicity of substrate
			Apo		3.1.3.4	
3.40.718.10	No	No	Mg/Zn	L1	1.1.1.42 1.1.1.85 1.1.1.262	Stabilizes charges and increases substrate acidity
			Apo		2.3.1.8

**Table 2:** Splitting events that separate ECs that use sites with different nuclearity (i.e. a different number of metal ions within the site). EC numbers associated to enzymes that use sites with different nuclearity e.g. in different organisms, were reported on the same line, with a list of all sites separated by “/”. See the caption to Table 1 for a further description of the contents in the various columns.

CATH code	Metal role conserved	Redox role	Ion(s)	EC diversity	EC(s)	Mechanism
3.40.630.10	Yes	No	Zn/Zn_2_	L2	3.5.1.18	Increases acidity and nucleophilicity of a water molecule and increases electrophilicity of substrate
			Zn_2_		3.4.11.10 3.4.11.4 3.5.1.14	
			Zn		3.4.17.1	
2.60.40.420	Yes	Yes	Cu	L2	1.7.2.1	Electron relay to substrate
			Cu_2_		1.10.3.2	
3.20.20.150	Yes	No	Zn/Mn	L1	4.2.1.8 5.3.1.22	Increases the acidity of the substrate
			Divalent_2_		5.3.1.5 5.3.1.14	
			Divalent_3_		3.1.21.2	
3.20.20.60	Yes	No	Mg_2_	L1	2.7.1.40	Stabilizes charges and increases substrate acidity
			Mg		5.4.2.9 2.7.3.9 2.1.2.11 2.7.9.2 4.1.3.1	
3.40.720.10	No	No	Mn_2_	L1	5.4.2.7	Increases nucleophilicity of a water molecule and electrophilicity of the phosphate moiety in the substrate
			Zn_2_		3.1.3.1	
			Mn/Co Ca/Mg		5.4.2.1 3.1.6.1 3.1.6.8 3.1.6.12 3.1.6.2	Stabilizes charges and increases electrophilicity of substrate
3.20.20.140	No	No	Zn_2_ Zn_2_/Fe_2_ Ni_2_	L1	3.5.2.3 3.4.13.19 3.5.1.25 3.5.4.2 3.5.1.5	Increases electrophilicity of the substrate and nucleophilicity of a second substrate or functional group.
			Zn		5.3.1.12	Increases acidity of substrate

**Table 3:** Splitting events that separate ECs that use different catalytic metal ions. See the caption to Table 1 for a further description of the contents in the various columns.

CATH code	Metal role conserved	Redox role	Ion(s)	EC diversity	EC(s)	Mechanism
3.40.718.10	Yes	No	Mg	L4	1.1.1.42 1.1.1.85	Stabilizes charges and increases substrate acidity
			Zn		1.1.1.262	
3.60.21.10	Yes	No	Fe,Mn	L2	3.1.3.2	Increases nucleophilicity of a water molecule and electrophilicity of the phosphate moiety in the substrate.
			Mn_2_		3.6.1.41	
			Divalent_2_		3.1.3.16 3.1.4.17 3.1.3.5 3.1.4.16	
3.40.50.1980	No	Yes	Fe	L2	1.18.6.1	Relays electrons
			Zn		1.1.1.23	Orients substrate in the cavity
2.60.120.10	No	Yes	Zn	L1	5.3.1.8 5.3.1.17	Stabilizes charges of substrates
			Cu/Fe		1.13.11.24 1.13.11.5	Stabilizes radical intermediate
			Mg		2.7.7.22	Stabilizes charges of substrates; increases electrophilicity of GTP
			Mn		4.1.1.2	Electron relay. Stabilizes radical
3.20.20.70	No	No	Mg	L1	4.1.1.85 4.2.1.24	Increases substrate acidity
			Zn Zn/Fe Zn/Co/Fe Zn/Mn		2.3.3.13 5.1.3.1 4.1.2.13 6.4.1.1	Increases substrate acidity
			Mn		2.5.1.54	Stabilizes charges of substrate and intermediate
3.40.720.10	Yes	No	Mn_2_	L1	5.4.2.7	Increases nucleophilicity of a water molecule and electrophilicity of the phosphate moiety in the substrate
			Zn_2_		3.1.3.1	
3.40.720.10	Yes	No	Mn/Co	L1	5.4.2.1	Stabilizes charges and increases electrophilicity of the substrate
			Ca/Mg		3.1.6.1 3.1.6.8 3.1.6.12 3.1.6.2	
1.20.1090.10	Yes	No	Zn/Fe	L1	1.1.1.1	Increases substrate acidity
			Zn		1.1.1.6 4.2.3.4	
			Mn/Zn		1.2.1.10	
			Fe		1.1.1.77	
3.10.180.10	No	Yes	Fe	L1	1.13.11.27 1.13.11.2	Binds O_2_. Reaction proceeds via oxidation of the iron(II)
			Zn/Ni		4.4.1.5	Stabilizes charges and increases substrate acidity
			Co		5.1.99.1	Stabilizes charges and increases substrate acidity
3.20.20.140	Yes	No	Zn	L2	3.5.2.3 3.4.13.19	Increases electrophilicity of the substrate and nucleophilicity of a second substrate or functional group.
			Zn/Fe		3.5.1.25 3.5.4.2	
			Ni		3.5.1.5	
3.60.15.10	No	Yes	Zn	L1	3.1.2.6 3.5.2.6 3.1.26.11	Increases nucleophilicity of a water molecule and electrophilicity of substrate
			Fe		1.7.1.14	Two iron ions transfer electrons to two NO molecules
3.90.850.10	Yes	No	Ca	L1	3.7.1.2 5.3.3.10	Stabilizes charges and increases electrophilicity of substrate
			Mg		4.1.1.68 4.2.1.80	
2.140.10.10	Yes	No	Ca	L1	1.1.5.2 1.1.99.8	Stabilizes charges and increases electrophilicity of substrate
			Any divalent		1.1.2.8	
			Zn		2.3.2.5

**Table 4:** Splitting events that separate ECs that bind the metal ion with different metal-binding ligands in the first sphere. See the caption to Table 1 for a description of the contents in the various columns.

CATH code	Metal role conserved	Redox role	Ion(s)	EC diversity	EC(s)	Change	Mechanism
3.90.245.10	Yes	No	Ca	L4	3.2.2.1	DDTD	Increases nucleophilicity of a water molecule.
					3.2.2.8	DDVD	
3.40.1190.10	Yes	No	Mg	L4	6.3.2.9 6.3.2.12	HSE	Stabilizes charges and increases electrophilicity of substrate
					6.3.2.8 6.3.2.10 6.3.2.13 6.3.2.17	THE	
3.20.20.150	Yes	No	Divalent_2_	L4	5.3.1.5	EEHDDDD	Increases the acidity of the substrate or of water
					5.3.1.14	EDHHDDD	
2.60.120.10	Yes	Yes	Cu/Fe	L4	1.13.11.5	H_EH	Stabilizes radical intermediate
					1.13.11.24	HHEH	
2.140.10.10	Yes	No	Ca	L3	1.1.5.2	EY	Stabilizes charges and increases electrophilicity of substrate
					1.1.99.8	END/E	
3.90.850.10	Yes	No	Mg	L2	4.1.1.68	EED	Stabilizes charges and increases electrophilicity of substrate
					4.2.1.80	EEE	
3.40.228.10	No	Yes	Mo-Fe	L2	1.2.1.2	C	Substrate binds to Mo(VI) and reduces it. Se-Cys typically one of the ligands
					1.7.99.4 1.6.6.9 1.7.2.3	S	Substrate binds to Mo(IV) and oxidizes it
3.40.50.620	Yes	No	Mg	L2	6.1.1.17 6.1.1.16 6.1.1.19 6.1.1.9	T	Stabilizes charges and increases electrophilicity of substrate
					6.3.1.5 6.3.3.4	DE	
3.40.630.10	Yes	No	Zn	L2	3.4.11.10 3.4.11.4	HHDED	Increases acidity and nucleophilicity of a water molecule and increases electrophilicity of substrate
					3.5.1.14	HDEEH	
3.20.20.70	Yes	No	Mg	L2	4.1.1.85	ED	Increases substrate acidity
					4.2.1.24	E	
3.40.50.2020	Yes	No	Mg	L2	2.4.2.9 2.4.2.14	DD	Stabilizes charges and increases electrophilicity of substrate
					2.7.6.1	H	
3.30.390.10	Yes	No	Mg	L1	4.2.1.11 4.2.1.113 5.5.1.1	DED	Orients substrate and stabilizes charges
					4.2.1.40	DEN	
					4.2.1.6	DEE	
3.30.470.20	Yes	No	Mg	L1	6.3.5.5	QEN	Stabilizes charges and increases electrophilicity of substrate
					6.3.2.4	DEN	
					6.3.4.13	_ EN	
					6.2.1.5	ND_	
					4.1.1.21	EE _	
3.40.50.300	Yes	No	Mg	L1	3.6.4.12 3.6.3.14 2.7.4.3	T	Stabilizes charges and increases electrophilicity of ATP
					3.6.4.13 2.7.7.7	D	
3.30.420.10	Yes	No	Mg	L1	3.1.26.4	DDN	Stabilizes charges and increases electrophilicity of substrate
					3.1.22.4	D _N	
					2.7.7.49	DDD	
3.40.50.1000	Yes	No	Mg	L1	3.1.3.18 3.1.3.3 3.1.3.5	DDD_	Stabilizes charges and increases electrophilicity of substrate
					3.11.1.1	_ _ DD	
					5.4.2.6	DDE _	
3.40.50.970	Yes	No	Mg	L1	2.2.1.6	DNH	Coordinates and orients substrate
					2.2.1.1	DNI	
					2.2.1.7	DNM	
					2.2.1.9	DNG	
					1.2.4.1	DNY	
1.10.600.10	Yes	No	Mg	L1	2.5.1.10 2.5.1.1	D_DD	Stabilizes charges and increases electrophilicity of substrate
					2.5.1.30	_ _DD	
					4.2.3.9	N_ DE	
					4.2.3.6	D_ D_	
					5.5.1.8	DD_D	
3.20.20.120	Yes	No	Mg/Mn	L1	4.2.1.11 4.2.1.113 5.5.1.1	DED	Stabilizes charges and increases substrate acidity
					4.2.1.40	DEN	
					4.2.1.6 5.1.2.2	DEE	
3.40.225.10	Yes	No	Zn	L1	4.1.2.17	EHHH	Increases substrate acidity
					4.1.2.19 4.2.1.109 4.1.2.13 5.1.3.4	_ HHH	
2.120.10.30	Yes	No	Ca	L1	1.1.5.2	GP	Stabilizes charges and increases substrate acidity
					3.1.1.17	TP	
1.20.1090.10	Yes	No	Zn	L1	1.1.1.6	DHH	Increases substrate acidity
					4.2.3.4	EHH	
3.20.20.150	Yes	No	Zn/Mn	L1	4.2.1.8	HC_H_ED	Increases the acidity of the substrate or of water
					5.3.1.22	EEHDDDD	

**Fig. 4:**
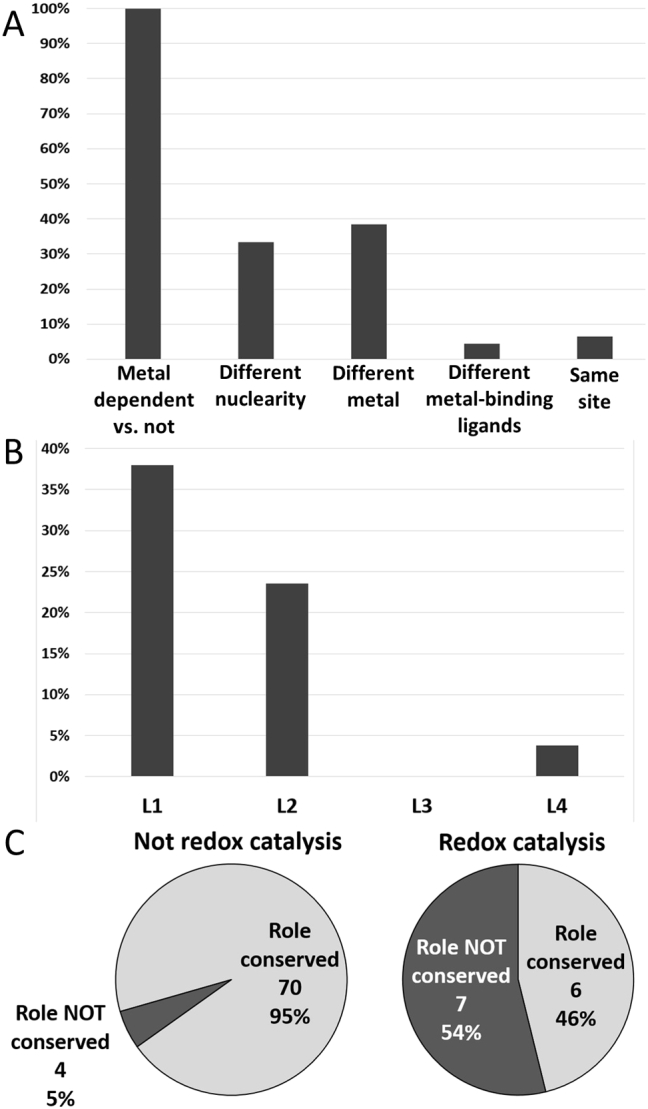
Statistics on the relationship between splitting events and change in the catalytic role of the metal site. The figure shows the percentage of splitting events for which the catalytic role of the metal ion is not conserved, separated by the type of structural change in the site (Panel A), and by the level of functional differentiation (Panel B). Panel C shows the same ratio for sites where the metal ion does not have a redox role (left) and for sites where the metal ion has a redox role (right), taking into account only superfamilies containing exclusively metalloenzymes.

Regardless of the occurrence of changes within the metal site, the role of the metal ion in the catalytic mechanism is generally maintained (about 85% of cases, i.e. 75 out of 88) (summarized in Table 1, Table 2, Table 3, Table 4). In contrast, when the catalytic metal binding is lost (in the remaining 13 instances) the mechanism of the enzyme necessarily undergoes a drastic modification (Table 4). Interestingly, we find that metals which participate in the reactions with redox roles are never lost. Changes in the role of the metal ion are more commonly associated to changes in the metal identity (Fig. 4A). These are also common when the site nuclearity varies, but the small number of cases examined might bias this observation. On the other hand, when the structure of the metal site is conserved and/or changes occur only in the protein residues in the first coordination sphere, the role of the metal ion within the reaction mechanism is generally conserved. Thus when the catalytic role of the metal ion changes, the functional differentiation is high i.e. diversity at EC level 1 and 2. In contrast, if the metal performs the same role, the functional differentiation is low i.e. diversity at EC level 4 (Fig. 4B).

When the metal ion is not involved in redox roles it is much more likely that the catalytic role is maintained (Fig. 4C). Out of 74 splitting events involving metal sites that did not transfer electrons to/from the substrate, in only 4 (5%) of cases did we observed different catalytic roles in the resulting subgroups. On the other hand, a different role of the metal in catalysis was observed for 7 out of 13 events (54%) involving at least one site where the metal ion had a redox role. Note that these data exclude superfamilies containing both metalloenzymes and non-metal dependent enzymes.

In the following, the relationships described above are detailed using selected relevant examples, divided according to the behaviour in terms of metal-binding properties.

#### 3.2.1 Splitting events separating enzymes that use metal ions from enzymes that do not require metals for catalysis

**Fig. 5:**
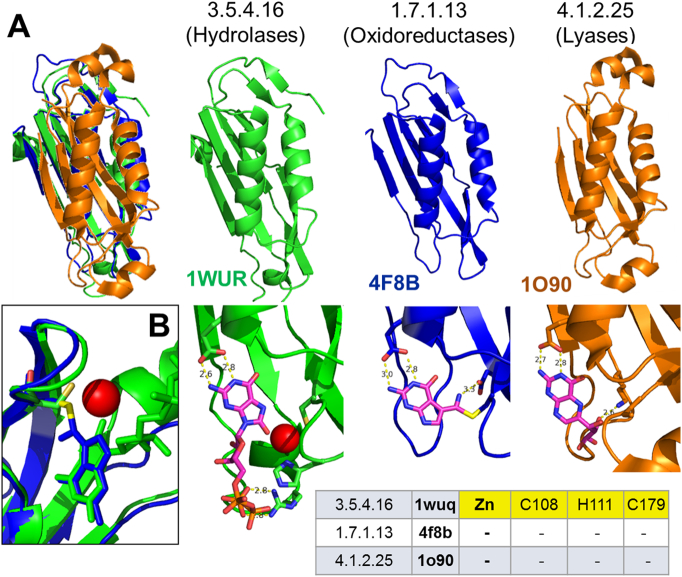
A superfamily (CATH 3.30.1130.10) containing enzymes with different EC numbers and gaining/losing a catalytic metal-binding site. The aligned protein structures (top), the aligned active sites with substrate-analogs bound (middle, the metal ion is depicted as a red sphere), and the structure-based alignment of the metal-binding ligands (bottom) are shown. The EC number of each enzyme is shown above the structure. (For interpretation of the references to color in this figure legend, the reader is referred to the web version of this article.)

This splitting event (Table 1) divides enzymes of the same superfamily that depend on catalytic metal ions from those that do not use metal ions to carry out their reaction mechanisms and have different EC numbers. Implicitly, the role of the metal ion is not conserved in all these thirteen cases identified because the metal ion role is completely lost in one of the enzyme pair. As an example, CATH superfamily 3.30.1130.10 includes the enzyme GTP cyclohydrolase IA (MM:0038), which is a zinc-dependent hydrolase (EC 3.5.4.16), and two related enzymes that do not rely on metal ions for catalysis, PreQ_0_ reductase (EC 1.7.1.13) and dihydroneopterin aldolase (EC 4.1.2.25). For all these enzymes, a 3D structure in complex with the substrate or a substrate analog is available (Fig. 5). The substrates are dicyclic compounds, either functionalized purines or pteridines. They contain a 2-amino-pyrimidine ring, where the second ring is either an imidazole or a pyrazine and it is this region that the enzyme targets. These three enzymes all interact in a very similar way with the common, unreactive 2-amino-pyrimidine ring, through the formation of H-bonds with the side chain of a conserved Glu. In GTP cyclohydrolase IA, the zinc(II) ion faces the imidazole ring of the substrate and activates a water molecule that acts as a nucleophile. The intermediate generated after the nucleophilic attack is proposed to remain bound to the zinc(II) ion on the basis of a structure with a substrate analog present [36], leading to formation of zinc-bound formate as one of the reaction products. Instead, PreQ_0_ reductase catalyzes the reduction of a nitrile group to a primary amine, whereas dihydroneopterin aldolase is a lyase that catalyzes the release of glycolaldehyde from the substrate, 7,8-dihydroneopterin. Intriguingly, PreQ_0_ reductase forms a covalent thioimide, a putative intermediate in the reaction, using the side chain of a Cys residue that is structurally equivalent to one of the zinc(II) ligands in GTP cyclohydrolase IA [37] (Fig. 5).

#### 3.2.2 Splitting events separating enzymes that use sites with different nuclearity (i.e. number of metal ions bound)

This relatively uncommon splitting event (only 7% of the total, Fig. 2A, Table 2), divides enzymes of the same superfamily that use a different number of metal ions in the catalytic center and have different EC numbers. This change is associated with a variation in the number of amino acid ligands recruited to form the site in order to provide the adequate number of ligands for all metal ions in the site. Variations in nuclearity occurred in only six of the superfamilies analyzed and, in our dataset, were always associated with large shifts in function (Fig. 3). The catalytic role of the metal site does not change in 67% of the cases. In the other 33%, the catalytic role of the site is either augmented by the additional contribution of the supplementary metal ion or changed altogether.

**Fig. 6:**
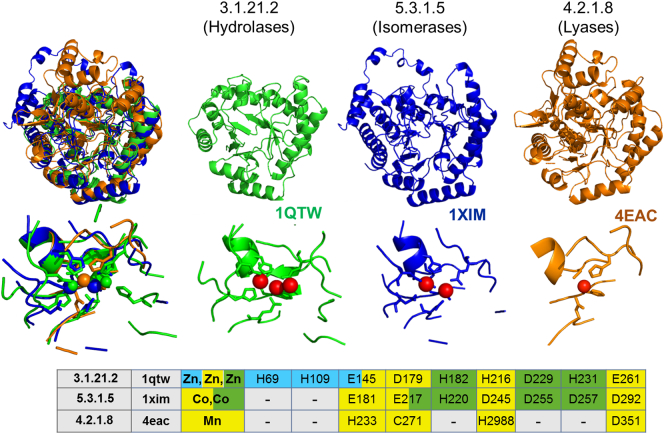
A superfamily (CATH 3.20.20.150) containing enzymes with different EC numbers and different nuclearity of the metal site. The aligned protein structures (top), the aligned metal site structures (middle, metal ions are depicted as red spheres), and the structure-based alignment of the metal-binding ligands (bottom, different colors indicate the ligands of individual metal ions) are shown. The EC number of each enzyme is reported above the structure. (For interpretation of the references to color in this figure legend, the reader is referred to the web version of this article.)

CATH superfamily 3.20.20.150 is an example of how a variable number of metal ions can ultimately provide the same contribution to the catalytic mechanism (Table 2). This superfamily contains enzymes that bind one, two or even three divalent cations to perform their function. Three divalent metal ions (either Zn^2 +^ or Mn^2 +^), are present in the active site of deoxyribonuclease IV [38] (EC 3.1.21.2, MM:M0011) (Fig. 6). The various isomerases that are present in this superfamily bind two (xylose isomerase, EC 5.3.1.5; L-rhamnose isomerase, EC 5.3.1.14, Table 2) or one divalent metal ion (hydroxypyruvate isomerase, EC 5.3.1.22). In all these systems, the site binds the organic substrate and activates it by increasing its acidity [39].

#### 3.2.3 Splitting events separating enzymes that use different catalytic metals

This splitting event (Table 3) divides enzymes of the same superfamily that use different metal ions for their catalysis and have different EC numbers. The coordination sphere of the metal ion is often modified to only a minor extent. These thirteen superfamilies featured the more profound levels of variation in EC number (Fig. 3). Even if the identity of the metal is different for different EC numbers, the catalytic role of the metal site does not change in 62% of the cases. Typically, when the role is unchanged, metal ions are involved in substrate activation through bond polarization caused by Lewis acidity, a common property among metal ions. In the remaining 38% of cases, the different metal ions play a different role in the catalytic mechanism because of their different redox properties.

**Fig. 7:**
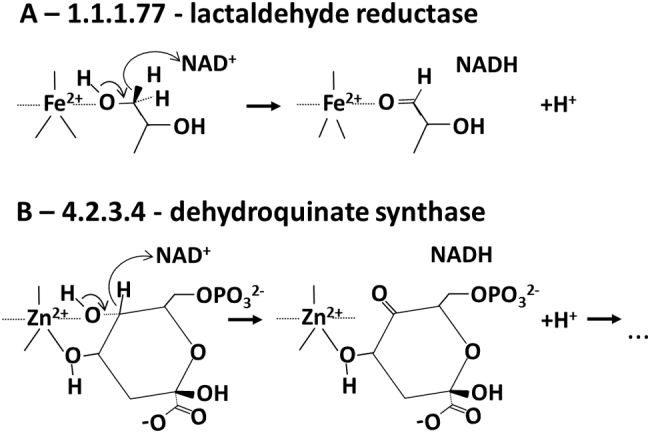
Comparison of the mechanisms of (A) lactaldehyde reductase and (B) dehydroquinate synthase. These two metal-dependent enzymes share the same fold and the binding site of the metal ion is located in corresponding positions in the two proteins. The enzymes are dependent on iron(II) and zinc(II), respectively, yet the reaction mechanism is analogous (see text for details).

An example where the different identity of the catalytic metal ion does not affect the enzyme mechanism is that of superfamily 1.20.1090.10, which includes family III metal-dependent polyol dehydrogenases [40], such as glycerol dehydrogenase (EC 1.1.1.6), lactaldehyde reductase (EC 1.1.1.77) or 1,3-propanediol dehydrogenase (EC 1.1.1.202), as well as dehydroquinate synthase (EC 4.2.3.4). The latter is a zinc(II)-dependent enzyme, whereas the polyol dehydrogenases typically depends on either zinc(II) or iron(II). All these enzymes share the same catalytic mechanism, regardless of the bound metal. In fact, the metal ion binds to the substrate, often in a bidentate manner, and increases the acidity of one of the hydroxyl groups. This favors proton dissociation followed by oxidation of the alcoholate to a carbonyl via the transfer of a hydride to NAD^+^. Thus, the different redox properties of zinc(II) and iron(II) do not matter: both metals are acting only as Lewis acids (Fig. 7). In dehydroquinate synthase the above process actually constitutes only the first step of the complex reaction catalyzed [41]. The oxidation of the alcohol is followed by beta-elimination of the phosphate group of the substrate and then by a reversal of the first step, as the ketone initially formed is reduced by NADH without the involvement of the zinc(II) ion.

A radically different behaviour is observed in the superfamily of metallo beta lactamases (CATH code: 3.60.15.10), where the identity of the catalytic metal determines the enzyme mechanism (Table 3). This family contains enzymes belonging to two distinct EC classes: hydrolases (glyoxalase II, EC 3.1.2.6; beta-lactamases, EC 3.5.2.6 and tRNase Z, EC 3.1.26.11) or oxidoreductases involved in the response to nitrosative and/or oxidative stress, such as rubredoxin:oxygen oxidoreductase [42]. While hydrolases are most commonly zinc(II)-dependent enzymes (only glyoxalase II is also active in the presence of metals other than zinc, such as iron(II) and manganese(II) [43]), oxidoreductases strictly require iron to perform the catalytic reaction. The metal-binding sites are located in corresponding positions, are structurally similar in the two groups of enzymes, and the metal cofactor is generally dinuclear (with the exception of type B2 metallo beta lactamases [44]). The metal ions bind directly to the substrate, correctly orienting it within the active site [45]. However, during the catalytic cycle the function of the metals is radically different in the hydrolases vs. the oxidoreductases. In the latter enzymes, each iron(II) ion transfers an electron to the substrate, thus providing two electrons in total upon forming a di-iron(III) site that is subsequently reduced by a FMNH_2_ molecule [46]. On the other hand, the zinc(II) site in the hydrolases is responsible for the activation of a water molecule for the nucleophilic attack on the substrate [47]. This type of mechanism is commonly observed in zinc-dependent hydrolases [14], [22], as zinc(II) is a strong Lewis acid. The only metal ligand that appears to change between the two classes of enzymes is a Glu residue in the di-iron(II) sites replacing a conserved His in the hydrolytic di-zinc(II) sites. It has been hypothesized that this Glu residue is able to suppress any possible hydrolytic cross-reactivity in the oxidoreductases [48].

#### 3.2.4 Splitting events separating enzymes that bind the same metal with different metal-binding ligands (different number or identity)

This splitting event (Table 4) divides enzymes of the same superfamily that use the same catalytic metal ion but have different first coordination spheres and have different EC numbers. Such variations generally affect one or two metal-binding ligands, and never more than half of all the protein ligands. This behaviour, which is the second most common in our dataset, is predominantly associated with the largest functional differentiation i.e. diversity at EC level 1 (Fig. 3, labelled as “Different metal-binding ligands”). More than 70% of these splitting events are associated with hard metal ions (e.g. Mg^2 +^, Ca^2 +^), which are less affected by changes in the identity of the ligands. Indeed, often the first coordination sphere of hard metal ion includes the backbone oxygen atoms of the protein residues rather than their side chains. The large majority of these splitting events (96%) maintain the catalytic role of the metal ion (Table 4) and the difference of the EC numbers is determined by other structural properties of the enzymes.

For example, CATH superfamily 1.10.600.10 contains isomerases, transferases and lyases. They all use three magnesium(II) ions to bind diphosphate-containing substrates. The interaction of the substrate with the three metals promotes the heterolysis of the C—O bond, leading to the formation of a carbocation and release of diphosphate. The rest of the reaction, which is different in the different enzymes, does not depend on the metal ion but depends on different properties in the structure of the different active sites [49], [50], [51].

The variation of first-sphere metal ligands is less common in sites that contain only donor atoms from protein side chains. Such coordination environments are typical of relatively soft metal ions, such as divalent transition metal ions (e.g. Zn^2 +^, Fe^2 +^). For these sites the metal-binding ligands are usually quite strictly conserved, a property which we previously showed to be useful for the prediction of metal-binding properties [52], [53]. Those protein ligands that are replaced within the superfamily are often conservatively substituted, so that the changes in the structure of the metal-binding site still leave the enzyme mechanism largely unaffected.

Superfamily 3.40.228.10 is the only example where the change in coordination sphere is associated with a change in the role of the metal cofactor in the catalytic mechanism. This superfamily includes, as an example, respiratory nitrate reductase (EC 1.7.99.4, MM:0276) and formate dehydrogenase (EC 1.2.1.2). The molybdenum ion that is directly responsible for electron exchange with the substrate has only one protein ligand: a cysteine in nitrate reductase [54] and a selenocysteine in formate dehydrogenase [55]. The coordination sphere of molybdenum includes a sulfide ion as an additional ligand in formate dehydrogenase compared to nitrate reductase. The different coordination environment stabilizes different oxidation states of the metal ion in the resting state of the two enzymes (+ 4 in nitrate reductase, + 6 in formate dehydrogenase), thus reversing the electron flow to/from the substrate (CATH superfamily 3.40.228.10, Table 4).

#### 3.2.5 Splitting events separating enzymes with the same metal-binding site

This splitting event (Table 5) divides enzymes of the same superfamily that have different EC numbers although they share the same properties of the catalytic metal site. In our dataset, this type of splitting event was associated with all possible levels of functional differentiation (i.e. L4, L3, L2, L1 in Fig. 3, label “Same site”). In the majority of cases (i.e. 93.4%), the catalytic role of the metal ion is maintained even if the EC number differs. In these examples the metal preserves its contribution to the mechanism whereas other changes in the protein environment affect other catalytic steps thereby leading to different EC numbers.

**Table 5:** Splitting events that separate ECs that use the same metal site. See the caption to Table 1 for a further description of the contents in the various columns.

CATH code	Metal role conserved	Redox role	Ion(s)	EC diversity	EC(s)	Mechanism
1.10.800.10	Yes	Yes	Fe	L4	1.14.16.1 1.14.16.2 1.14.16.4	Transfers electrons to O_2_ from substrates via a ferryl-oxo intermediate
3.10.170.10	Yes	No	Zn	L4	3.4.24.28 3.4.24.29 3.4.24.25	Increases nucleophilicity of a water molecule and electrophilicity of the scissile amide bond.
3.40.120.10	Yes	No	Mg	L4	5.4.2.10 5.4.2.8 5.4.2.2	Stabilizes charges and increases electrophilicity of substrate
3.40.1190.10	Yes	No	Mg	L4	6.3.2.8 6.3.2.10 6.3.2.13 6.3.2.17	Stabilizes charges and increases electrophilicity of substrate
3.40.1190.10	Yes	No	Mg	L4	6.3.2.9 6.3.2.12	Stabilizes charges and increases electrophilicity of substrate
1.10.600.10	Yes	No	Mg	L4	2.5.1.10 2.5.1.1	Stabilizes charges and increases electrophilicity of substrate
3.90.228.20	Yes	No	Mg	L4	4.1.1.49 4.1.1.32	Stabilizes charges and increases electrophilicity of substrate
3.30.70.1230	Yes	No	Mg	L4	4.6.1.1 4.6.1.2	Stabilizes charges and increases electrophilicity of substrate
3.40.140.10	Yes	No	Zn	L4	3.5.4.5 3.5.4.2 3.5.4.12	Increases acidity and nucleophilicity of a water molecule
3.40.720.10	Yes	No	Ca/Mg	L4	3.1.6.1 3.1.6.8 3.1.6.12 3.1.6.2	Stabilizes charges and increases electrophilicity of substrate
3.40.630.10	Yes	No	Zn	L4	3.4.11.10 3.4.11.4	Increases acidity and nucleophilicity of a water molecule and increases electrophilicity of substrate
3.20.20.190	Yes	No	Ca	L4	3.1.4.11 3.1.4.46	Stabilizes charges and increases electrophilicity of substrate
3.40.50.720	Yes	No	Mg/Mn	L4	1.1.1.38 1.1.1.39 1.1.1.40	Stabilizes charges and increases substrate acidity
3.40.50.2020	Yes	No	Mg	L4	2.4.2.9 2.4.2.14	Stabilizes charges and increases electrophilicity of substrate
3.40.50.1000	Yes	No	Mg	L4	3.1.3.18 3.1.3.3 3.1.3.5	Stabilizes charges and increases electrophilicity of substrate
3.90.180.10	Yes	No	Zn	L4	1.1.1.1 1.1.1.14 1.1.1.284 1.1.1.103	Increases substrate acidity
3.40.140.10	Yes	No	Zn	L4	3.5.4.5 3.5.4.2 3.5.4.12	Increases acidity and nucleophilicity of a water molecule
1.10.606.10	Yes	No	V	L4	1.11.1.10 1.11.1.18	Stabilizes charges and increases electrophilicity of substrate
3.40.718.10	Yes	No	Mg	L4	1.1.1.42 1.1.1.85	Stabilizes charges and increases substrate acidity
3.10.180.10	Yes	Yes	Fe	L4	1.13.11.2 1.13.11.27	Binds O_2_. Reaction proceeds via oxidation of the iron(II)
2.60.120.10	Yes	No	Zn	L3	5.3.1.8 5.3.1.17	Stabilizes charges of substrate
3.20.20.140	Yes	No	Zn/Fe	L3	3.5.1.25 3.5.4.2	Increases electrophilicity of the substrate and nucleophilicity of a second substrate or functional group.
1.10.390.10	Yes	No	Zn	L3	3.4.11.2 3.4.24.28 3.4.24.29	Increases acidity and nucleophilicity of a water molecule and electrophilicity of the scissile amide bond.
1.10.510.10	Yes	No	Mg	L3	2.7.11.1 2.7.10.1 2.7.10.2 2.7.11.17	Stabilizes charges and increases electrophilicity of substrate
3.90.380.10	Yes	Yes	Fe	L3	1.14.12.19 1.14.12.18 1.14.12.10 1.14.13.82 1.14.12.1	It catalyzes the transfer of electrons to O_2_ from substrates via a high-valent intermediate
3.60.21.10	Yes	No	Divalent	L3	3.1.3.16 3.1.4.17 3.1.3.5 3.1.4.16	Increases nucleophilicity of a water molecule and electrophilicity of the phosphate moiety in the substrate
1.10.575.10	Yes	No	Zn	L3	3.1.4.3 3.1.30.1	Increases nucleophilicity and electrophilicity of the substrates
3.30.479.10	Yes	No	Zn	L2	4.2.3.12 4.1.2.50	Stabilizes charges and increases substrate acidity
3.20.20.140	Yes	No	Zn	L2	3.5.2.3 3.4.13.19	Increases electrophilicity of the substrate and nucleophilicity of a second substrate or functional group.
3.60.15.10	Yes	No	Zn	L2	3.1.2.6 3.5.2.6 3.1.26.11	Increases nucleophilicity of a water molecule and electrophilicity of substrate
1.10.1280.10	Yes	No	Cu	L2	1.14.18.1 1.10.3.1	Monophenolase and diphenolase activity
2.60.120.330	Yes	Yes	Fe	L2	1.14.11.6 1.14.17.4 1.14.11.9 1.21.3.1	Transfers electrons to O_2_ from substrates via a ferryl-oxo intermediate
1.10.560.10	Yes	No	Mg	L1	2.7.1.150 3.6.1.3 3.6.4.9 6.3.5.5	Stabilizes charges and increases electrophilicity of substrate
1.50.10.10	Yes	No	Ca	L1	3.2.1.28 3.2.1.4 5.1.3.8 2.4.1.8	Coordinates and orients substrate
3.30.565.10	Yes	No	Mg	L1	2.7.13.3 5.99.1.3	Stabilizes charges and increases electrophilicity of substrate
3.40.50.280	No	Yes	Co	L1	2.1.1.13	The Co—C bond of methylcobalamin is cleaved heterolytically
					5.4.99.1 5.4.99.2	The Co—C bond of adenosylcobalamin is cleaved homolytically
3.60.120.10	Yes	No	Mg	L1	4.1.3.27 5.4.4.2 2.6.1.85	Stabilizes charges
3.90.850.10	Yes	No	Ca	L1	3.7.1.2 5.3.3.10	Stabilizes charges and increases electrophilicity of substrate
3.30.390.10	Yes	No	Mg	L1	4.2.1.11 4.2.1.113 5.5.1.1	Orients substrate and stabilizes charges
3.20.20.120	Yes	No	Mg	L1	4.2.1.11 4.2.1.113 5.5.1.1	Stabilizes charges and increases substrate acidity
3.20.20.120	Yes	No	Mg	L1	4.2.1.6 5.1.2.2	Stabilizes charges and increases substrate acidity
3.40.50.300	Yes	No	Mg	L1	3.6.4.12 3.6.3.14 2.7.4.3	Stabilizes charges and increases electrophilicity of ATP
3.40.50.300	Yes	No	Mg	L1	3.6.4.13 2.7.7.7	Stabilizes charges and increases electrophilicity of ATP
1.10.630.10	No	Yes	Fe-heme	L1	1.14.14.1 1.14.13.70	Monooxygenation occurs via a ferryl-porphyrin cation radical
					4.2.1.92	A protonated ferryl intermediate is formed not suitable for MO
3.20.20.60	No	No	Mg	L1	2.7.3.9 2.1.2.11 2.7.9.2 4.1.3.1	Stabilizes charges and increases substrate acidity
					5.4.2.9	Promotes heterolysis of a P—O bond
3.40.225.10	Yes	No	Zn	L1	4.1.2.19 4.2.1.109 4.1.2.13 5.1.3.4	Increases substrate acidity

A change in the catalytic role of the metal ion occurs only in three superfamilies. A first example is the CATH superfamily 3.40.50.280, which contains enzymes with different cobalamin-based cofactors (Table 5). Each enzyme in the superfamily bind the cofactor with a single histidine ligand. A second example is the CATH superfamily 1.10.630.10 that contains Fe-heme dependent enzymes with the cytochrome P_450_ fold. Among these in the so-called CYP74 subfamily the electronic properties of the catalytic iron ion are tuned so as to avoid the creation of the crucial ferryl-porphyrin cation radical normally formed in cytochromes P_450_ skipping directly to a protonated ferryl intermediate [56], [57]. In cytochromes P_450_ the latter would hydroxylate the substrate radical, whereas in the CYP74 subfamily it participates in electron transfer or oxygen rebound, depending on the specific enzyme. The last case is the CATH superfamily 3.20.20.60, which includes Mg^2 +^-dependent enzymes. In most of the enzymes of this superfamily, the metal ion is involved in activating the substrate by increasing its acidity; in contrast in phosphoenolpyruvate mutase (EC 5.4.2.9), another member of this family, the magnesium ion promotes heterolysis of a P—O bond.

## 4 Discussion

The role of catalytic metal ions within the overall enzyme mechanism is one of the specific interests of bioinorganic chemistry. Furthermore, it has been also demonstrated that almost 40% of the enzymes with an available structure use a metal ion to carry out their reaction mechanism [14]. In this work we analyzed 64 CATH superfamilies containing metal-dependent enzymes to systematically investigate the effects of specific changes in the metal-containing active site on the function of metalloenzymes. The approach implemented relies on the structural and functional information available from specialized databases. Within this context, the current dataset provides an opportunity to investigate the interplay between the contributions to the catalytic mechanism of the protein framework and the metal site. Indeed, it is the combination of these two aspects that ultimately determines the evolution of the enzyme chemistry summarized by the EC number. The present analysis of metalloenzymes on a per-superfamily basis, i.e. within a constant global protein fold, ensures that there is an evolutionary relationship among the systems compared [10], [11], [58], and thus can provide useful insight into this interplay. Even if the present dataset is too small to derive statistically meaningful conclusions, a few clear trends emerge as discussed below.

It is important to note that the pipeline does not attempt to reveal the evolutionary direction of the changes we observe. We have developed a way to group the type of differences in function observed with the concomitant changes in metal binding and site. It is also important to note that mostly throughout evolution function is maintained, but in this study we have chosen to focus particularly on those examples where function has changed, caused by changes in both the metal binding and the larger protein framework. Here we simply observe the types of changes in enzyme function which occur in metalloenzymes and their frequency in the current dataset (which is limited to just 65 domain superfamilies in CATH, since we need to know function, mechanism and structures for this analysis). To understand the timelines of these changes requires a more detailed phylogenetic analysis, based on many protein sequences to produce robust phylogenetic trees and allow ancestral protein sequence reconstructions.

Evolution tends to strictly conserve the site of the catalytic metal ion. Indeed, in 45% of cases, changes of the EC numbers of the members of any given superfamily do not correspond to changes in the properties of the metal-binding site (Fig. 2A). This is unsurprising when the superfamily is characterized by low functional diversification, i.e. EC numbers changes at the third and fourth levels (L3 and L4). However, there are superfamilies with high functional diversity whose members all maintain the same metal site (Fig. 3). This demonstrates that the evolution of the protein moiety is as important as events affecting the metal site to ultimately determine the function of a metalloenzyme. In these particular systems, the metal is typically involved in substrate binding, determining its orientation and activation within the active site. These can be the first steps of the overall catalytic mechanism, common to various enzymes of a given superfamily regardless of their specific function. The specific reactivity of these metalloenzymes is thus determined not by rearranging the metal coordination sphere but through the interaction of the protein matrix with the (metal-bound) substrate, i.e. second sphere interactions, by mutating other residues involved in the catalytic mechanism, or by binding additional cofactors and/or co-substrates.

Any variation in the metal site properties, even variations as small as changes in the first coordination sphere of the metal ion, are very likely associated with high functional differentiation (Fig. 3). To some extent this may also reflect the many sequence variations throughout the protein moiety, only some of which affect the metal binding site; by the time advantageous mutations have appeared at the metal site, several others will probably have accumulated along the enzyme chain. The combination of these two phenomena causes the observed functional differentiation.

Our results suggest that the role of the metal cofactor in the catalytic mechanism of the enzyme is more stable (evolves more slowly) than the overall enzyme chemistry. Indeed, the contribution of the metal cofactor to the mechanism is essentially the same in more than 85% of the observed examples (excluding cases where some superfamily members loose metal-binding capability altogether). The catalytic role of the metal ion is more likely to change when the functional differentiation within the superfamily is high (Fig. 4B). When the metal site properties are conserved or variation just occurs in the first coordination sphere, the metal role is conserved in almost all cases (Fig. 4A). The only exceptions involve redox-active metal ions that participate in the reaction by exchanging electrons with the substrate. These often change their role. Larger scale structural changes in the metal site, such as metal replacement or changes in site nuclearity, increase the likelihood of varying the catalytic role of the metal (Fig. 4A). Conservation of the catalytic role is more likely when metal ions are not involved in redox catalysis (Fig. 4C). Indeed, in these reaction mechanisms, metal ions contribute to the catalysis mainly via electrostatic interactions and/or direct coordination to the substrate. In turn, these may stabilize charged intermediates or polarize reactive bonds. This is principally the situation for the hardest metal ions such as magnesium(II) or calcium(II), but also for several zinc(II)-enzymes. In these metalloenzyme superfamilies, the mainly electrostatic nature of the metal contribution to catalysis makes it possible for the metal to be replaced by an appropriate network of hydrogen bond interactions or by positively charged amino acids, such as in class I vs. class II fructose-biphosphate aldolase [59].

For metal ions that participate in the reaction mechanism by directly donating/accepting electrons to/from the substrate (redox catalysis) the situation is more nuanced. When catalysis relies on the fine-tuning of the reduction potential of the metal ion, changes around the metal site may shift the potential enough to hamper or reroute enzymatic activity [60]. A well characterized example is that of the vicinal oxygen chelate fold (CATH superfamily 3.10.180.10), whose members include a variety of metal configurations. Multiple divalent metals may originally have acted in catalysis merely as Lewis acids, evolving first to exploiting one or two metals for the catalysis of redox reactions that did not involve oxidation changes at the metal ion, and then to be specific for iron(II) once the metal ion got directly involved in redox chemistry [61]. Another scenario can occur when the electronic interaction between the metal and the substrate determines the outcome of the reaction, and it becomes possible that a different selection of the substrate by the protein matrix or the stabilization of different electronic states during catalysis alter the reaction mechanism between members of the same superfamily [62], as exemplified here for the cytochrome P_450_ superfamily [57], [63] (Table 5). When the contribution to the catalysis does not actually involve electron transfer to/from the substrate, the role of redox-active metals is most often conserved, even upon metal substitution, (see Fig. 7). Some metalloenzymes in vivo can bind different metals in a promiscuous manner or depending on environmental availability. This indicates how the protein fold, starting with substrate selection, can indeed steer metal reactivity [64], [65], [66]. A completely different scenario, which we did not address here, is the one in which different protein folds bind different metal ions to achieve the same catalytic reaction [15]. It is also important to keep in mind that we analyzed only physiologically relevant, active enzyme forms, with supporting 3D structural data available [22]. We thus excluded all cases in which metal replacements have been tested only in vitro or lead to enzyme inactivation.

In summary, the metal-binding sites in metalloenzymes tend to provide similar contributions to the catalytic mechanism within each superfamily. This is particularly true for metal ions not participating in redox catalysis (Fig. 4C). Indeed, the field of metalloprotein design has extensively exploited this general property, by crafting predesigned sites in existing or de novo designed folds [67]. The scenario is different for sites with redox active metals, especially when electronic interactions with the substrate or reaction intermediates play an important role in catalysis. Such interactions can be very sensitive to local structural rearrangements thus making the contribution of the metal site to the enzyme mechanism more prone to change during evolution. The latter consideration suggests that the evolution of a non-metal-dependent enzyme into a metalloenzyme is a path less likely to occur if the redox activity and properties of the metal site become crucial for catalysis. Notably, in all the 13 superfamilies containing both metal-dependent and not-metal-dependent-enzymes (Table 1) the catalytic metal is never involved in redox reactions, consistent with the idea that proteins can replace the contribution of metals such as magnesium(II) or zinc(II) more easily than that of redox metals. On the other hand, with a constant contribution by the metal cofactor to the catalytic mechanism, the protein matrix dominates the selection of the substrate and ultimately determines the end point of the reaction. It is mainly the largest structural changes of the site that are likely to impact significantly on the catalytic mechanism, possibly even surpassing the effect of the evolution of the rest of the protein sequence. Metal gain/loss, which is an extreme scenario yet easily possible with rather few sequence changes, is one of the most common mechanisms likely to change function during evolution observed to date.

## Abbreviations

ATP: adenosine triphosphate
CATH: Class, Architecture, Topology and Homologous superfamily classification of protein domains
EC: Enzyme Commission
EG: equistructural group
FeMoco: a cluster with composition Fe_7_MoS_9_C that is the primary cofactor of nitrogenases
GTP: guanosine triphosphate
GYCH-I: GTP cyclohydrolase I
MACiE: the database of Mechanism, Annotation and Classification in Enzymes
Metal-MACiE: the Metal-dependent subset of the MACiE database
MetalPDB: the Metal-binding subset of the Protein Data Bank
MetalS2: Metal Sites Superposition tool
MetalS3: Metal Sites Similarity Search tool
MM: Metal-MACiE database entry
PDB: Protein Data Bank

The following is the supplementary data related to this article.

Supplementary Table S1 Superfamilies without splitting events. This table lists superfamilies that contain enzymes all associated to a single EC number. Supplementary Table S1

## References

[1] Martinez C.S., Rahman S.A., Furnham N., Thornton J.M. (2015). Biophys. J..

[2] Dayhoff M.O., Barker W.C., Hunt L.T. (1983). Methods Enzymol..

[3] Holm L., Sander C. (1999). Nucleic Acids Res..

[4] Levitt M. (2009). Proc. Natl. Acad. Sci. U. S. A..

[5] Lo Conte L., Ailey B., Hubbard T.J., Brenner S.E., Murzin A.G., Chothia C. (2000). Nucleic Acids Res..

[6] Orengo C.A., Jones D.T., Thornton J.M. (1994). Nature.

[7] Orengo C.A., Michie A.D., Jones S., Jones D.T., Swindells M.B., Thornton J.M. (1997). Structure.

[8] Bartlett G.J., Borkakoti N., Thornton J.M. (2003). J. Mol. Biol..

[9] Glasner M.E., Gerlt J.A., Babbitt P.C. (2006). Curr. Opin. Chem. Biol..

[10] Brown S.D., Babbitt P.C. (2014). J. Biol. Chem..

[11] Galperin M.Y., Koonin E.V. (2012). J. Biol. Chem..

[12] Furnham N., Sillitoe I., Holliday G.L., Cuff A.L., Laskowski R.A., Orengo C.A. (2012). PLoS Comput. Biol..

[13] Furnham N., Sillitoe I., Holliday G.L., Cuff A.L., Rahman S.A., Laskowski R.A. (2012). Nucleic Acids Res..

[14] Andreini C., Bertini I., Cavallaro G., Holliday G.L., Thornton J.M. (2008). J. Biol. Inorg. Chem..

[15] Valdez C.E., Smith Q.A., Nechay M.R., Alexandrova A.N. (2014). Acc. Chem. Res..

[16] Maret W., Li Y. (2009). Chem. Rev..

[17] Andreini C., Bertini I., Cavallaro G. (2011). PLoS ONE.

[18] Choi M., Davidson V.L. (2011). Metallomics.

[19] Lee Y.M., Lim C. (2011). J. Am. Chem. Soc..

[20] Bertini I., Gray H.B., Stiefel E.I., Valentine J.S. (2006). Biological Inorganic Chemistry.

[21] Frausto da Silva J.J.R., Williams R.J.P. (2001). The Biological Chemistry of the Elements: The Inorganic Chemistry of Life.

[22] Andreini C., Bertini I., Cavallaro G., Holliday G.L., Thornton J.M. (2009). Bioinformatics.

[23] Andreini C., Cavallaro G., Lorenzini S., Rosato A. (2013). Nucleic Acids Res..

[24] Rosato A., Valasatava Y., Andreini C. (2016). Int. J. Mol. Sci..

[25] Vallee B.L., Williams R.J. (1968). Proc. Natl. Acad. Sci. U. S. A..

[26] Christianson D.W. (1991). Adv. Protein Chem..

[27] Williams R.J. (1995). Eur. J. Biochem..

[28] Sillitoe I., Lewis T.E., Cuff A., Das S., Ashford P., Dawson N.L. (2015). Nucleic Acids Res..

[29] Zhang Y., Skolnick J. (2005). Nucleic Acids Res..

[30] Berman H., Henrick K., Nakamura H. (2003). Nat. Struct. Biol..

[31] Martin A.C. (2004). Bioinformatics.

[32] Barthelmes J., Ebeling C., Chang A., Schomburg I., Schomburg D. (2007). Nucleic Acids Res..

[33] Andreini C., Cavallaro G., Rosato A., Valasatava Y. (2013). J. Chem. Inf. Model..

[34] Valasatava Y., Rosato A., Cavallaro G., Andreini C. (2014). J. Biol. Inorg. Chem..

[35] Sankaran B., Bonnett S.A., Shah K., Gabriel S., Reddy R., Schimmel P. (2009). J. Bacteriol..

[36] Tanaka Y., Nakagawa N., Kuramitsu S., Yokoyama S., Masui R. (2005). J. Biochem..

[37] Chikwana V.M., Stec B., Lee B.W., Crecy-Lagard V., Iwata-Reuyl D., Swairjo M.A. (2012). J. Biol. Chem..

[38] Tsutakawa S.E., Shin D.S., Mol C.D., Izumi T., Arvai A.S., Mantha A.K. (2013). J. Biol. Chem..

[39] Martinez C.S., Furnham N., Rahman S.A., Sillitoe I., Thornton J.M. (2014). Curr. Opin. Struct. Biol..

[40] Ruzheinikov S.N., Burke J., Sedelnikova S., Baker P.J., Taylor R., Bullough P.A. (2001). Structure.

[41] Carpenter E.P., Hawkins A.R., Frost J.W., Brown K.A. (1998). Nature.

[42] Frazao C., Silva G., Gomes C.M., Matias P., Coelho R., Sieker L. (2000). Nat. Struct. Biol..

[43] Campos-Bermudez V.A., Leite N.R., Krog R., Costa-Filho A.J., Soncini F.C., Oliva G. (2007). Biochemistry.

[44] Hu Z., Gunasekera T.S., Spadafora L., Bennett B., Crowder M.W. (2008). Biochemistry.

[45] Spencer J., Read J., Sessions R.B., Howell S., Blackburn G.M., Gamblin S.J. (2005). J. Am. Chem. Soc..

[46] Silaghi-Dumitrescu R., Kurtz D.M., Ljungdahl L.G., Lanzilotta W.N. (2005). Biochemistry.

[47] de la Sierra-Gallay Li, Pellegrini O., Condon C. (2005). Nature.

[48] Schilling O., Vogel A., Kostelecky B., Natal D.L., Spemann D., Spath B. (2005). Biochem. J..

[49] Hosfield D.J., Zhang Y., Dougan D.R., Broun A., Tari L.W., Swanson R.V. (2004). J. Biol. Chem..

[50] Rynkiewicz M.J., Cane D.E., Christianson D.W. (2001). Proc. Natl. Acad. Sci. U. S. A..

[51] Whittington D.A., Wise M.L., Urbansky M., Coates R.M., Croteau R.B., Christianson D.W. (2002). Proc. Natl. Acad. Sci. U. S. A..

[52] Andreini C., Bertini I., Rosato A. (2009). Acc. Chem. Res..

[53] Andreini C., Bertini I., Cavallaro G., Decaria L., Rosato A. (2011). J. Chem. Inf. Model..

[54] Arnoux P., Sabaty M., Alric J., Frangioni B., Guigliarelli B., Adriano J.M. (2003). Nat. Struct. Biol..

[55] Raaijmakers H.C., Romao M.J. (2006). J. Biol. Inorg. Chem..

[56] Stumpe M., Feussner I. (2006). Phytochem. Rev..

[57] Lee D.S., Nioche P., Hamberg M., Raman C.S. (2008). Nature.

[58] Todd A.E., Orengo C.A., Thornton J.M. (2001). J. Mol. Biol..

[59] Nagano N., Orengo C.A., Thornton J.M. (2002). J. Mol. Biol..

[60] Malmström B.G. (1994). Eur. J. Biochem..

[61] He P., Moran G.R. (2011). J. Inorg. Biochem..

[62] Sparta M., Valdez C.E., Alexandrova A.N. (2013). J. Mol. Biol..

[63] Das A., Grinkova Y.V., Sligar S.G. (2007). J. Am. Chem. Soc..

[64] He M.M., Clugston S.L., Honek J.F., Matthews B.W. (2000). Biochemistry.

[65] Emerson J.P., Kovaleva E.G., Farquhar E.R., Lipscomb J.D., Que L. (2008). Proc. Natl. Acad. Sci. U. S. A..

[66] Mandelli F., Franco Cairo J.P., Citadini A.P., Buchli F., Alvarez T.M., Oliveira R.J. (2013). Lett. Appl. Microbiol..

[67] Yu F.T., Cangelosi V.M., Zastrow M.L., Tegoni M., Plegaria J.S., Tebo A.G. (2014). Chem. Rev..

