# Supplementary material for: To what extent do structural changes in catalytic metal sites affect enzyme function?
Source: J Inorg Biochem. 2018 Feb;179:40–53. doi: 10.1016/j.jinorgbio.2017.11.002 (PMC5760197; doi:10.1016/j.jinorgbio.2017.11.002)
Supplement: Supplementary Table S1 — Superfamilies without splitting events. This table lists superfamilies that contain enzymes all associated to a single EC number. [file mmc1.docx]

**Supplementary Table S1: Superfamilies without splitting events.** This table lists superfamilies that contain enzymes all associated to a single EC number.

| **CATH code** | **Metal role**  **conserved** | **Ion(s)** | **EC(s)** | **Mechanism** |
| --- | --- | --- | --- | --- |
| 3.40.50.1260 | YES | Mg | 2.7.2.3 | Stabilizes charges and increases electrophilicity of substrate |
| 1.20.90.10 | YES | Ca | 3.1.1.4 | Stabilizes charges and increases electrophilicity of substrate |
| 2.40.50.90 | YES | Ca | 3.1.31.1 | Stabilizes charges and increases electrophilicity of substrate |
| 2.60.40.200 | YES | Cu | 1.15.1.1 | Accepts and donates one electron from/to the substrate |
| 3.90.470.20 | YES | Mg/Mn | 2.7.8.7 | Increases substrate acidity |
| 3.10.200.10 | YES | Zn | 4.2.1.1 | Increases substrate nucleophilicity and acidity |
| 3.90.45.10 | YES | Fe | 3.5.1.88 | Increases substrate nucleophilicity and acidity |
| 3.30.1130.10 | YES | Zn | 3.5.4.16 | Increases acidity and nucleophilicity of a water molecule |
